# The Impact of Sperm Metabolism during* In Vitro* Storage: The Stallion as a Model

**DOI:** 10.1155/2016/9380609

**Published:** 2016-01-12

**Authors:** Zamira Gibb, Robert J. Aitken

**Affiliations:** Priority Research Centre for Reproductive Science, Discipline of Biological Sciences, Faculty of Science and IT, University of Newcastle, Callaghan, NSW 2308, Australia

## Abstract

*In vitro* sperm storage is a necessary part of many artificial insemination or* in vitro* fertilization regimes for many species, including the human and the horse. In many situations spermatozoa are chilled to temperatures between 4 and 10°C for the purpose of restricting the metabolic rate during storage, in turn, reducing the depletion of ATP and the production of detrimental by-products such as reactive oxygen species (ROS). Another result of lowering the temperature is that spermatozoa may be “cold shocked” due to lipid membrane phase separation, resulting in reduced fertility. To overcome this, a method of sperm storage must be developed that will preclude the need to chill spermatozoa. If a thermally induced restriction-of-metabolic-rate strategy is not employed, ATP production must be supported while ameliorating the deleterious effects of ROS. To achieve this end, an understanding of the nature of energy production by the spermatozoa of the species of interest is essential. Human spermatozoa depend predominantly on glycolytic ATP production, producing significantly less ROS than oxidative phosphorylation, with the more efficient pathway predominantly employed by stallion spermatozoa. This review provides an overview of the implications of sperm metabolism for* in vitro* sperm storage, with a focus on ambient temperature storage in the stallion.

## 1. Introduction

Horses are selected for breeding on the basis of pedigree and athletic performance as opposed to reproductive traits and therefore, like humans, are not subjected to selection pressure for fertility. Reproductive fitness traits are heritable [1], and the practice of circumventing subfertility through the use of assisted reproductive technologies (ART), because it places no importance on reproductive fitness in the selection of breeding animals or partners, has resulted in equine and human populations with significantly lower per cycle conception rates than other species [1–3]. As artificial insemination (AI) is a widely utilised tool in modern horse reproduction [4], with around 90% of Standardbred and Hanoverian foals being produced via AI of chilled or cryopreserved stallion spermatozoa [2, 5], this animal model provides an excellent source of information about the influence of cell metabolism on the storage of male gametes. For its part, the use of AI brings a number of advantages, such as the prevention and control of disease through the eradication of direct male to female contact [6], an increased rate of genetic gain through the importation of new genetics and the preservation of spermatozoa for later use in case of death or infertility.

## 2. Sperm Metabolism

Spermatozoa are highly specialised mammalian cells, playing the vital roles of paternal DNA delivery and activation of the oocyte following fertilisation. The site of sperm deposition (in the vagina for the human and the uterus for the horse) is physically removed from the site of fertilisation (the oviduct). While a proportion of sperm transport is facilitated by uterine contractions, the spermatozoa must in themselves be sufficiently motile to traverse the uterotubal junction prior to oviduct binding and to locate the egg following ovulation. In addition, spermatozoa must undergo a process called capacitation for the final maturational changes that are required to allow them to fertilise the oocyte. This process involves extreme membrane remodelling and the hyperactivation of motility and, as such, is a highly energy-dependent process [7].

The process of spermatogenesis requires extensive remodelling of a conventional spherical cell to become one of the most highly specialised and morphologically distinct cells in the body. During this transformation, the DNA in the sperm nucleus reaches the physical limits of compaction to achieve a quasicrystalline state [8]. This extreme compaction requires the removal or resorption of most of the cytoplasm, at the same time removing the majority of the organelles (such as the endoplasmic reticulum, ribosomes, and Golgi apparatus) that are responsible for the regulation of metabolism in somatic cells. The result of this drastic modification is that spermatozoa are left both translationally silent and depleted of intracellular enzymes and energy reserves such as fat droplets, yolk granules, and glycogen. For this reason, spermatozoa are highly dependent on their immediate extracellular environment for both the enzymatic activities that would normally be conducted intracellularly and the supply of energy substrates [9]. In somatic cells, the array of enzymes involved in protecting spermatozoa against oxidative stress would also be housed intracellularly within the cytoplasm. Spermatozoa, on the other hand, depend upon epididymal and seminal fluids to provide the richest and most diverse combination of antioxidants in the body, including several antioxidants that are unique to the male reproductive tract [10, 11].

As with somatic cells, the predominant metabolic pathways that spermatozoa use to produce ATP are glycolysis and oxidative phosphorylation (OXPHOS) [12]. The enzymes necessary for glycolysis are primarily associated with the fibrous sheath located in the principal piece of the tail. In contrast, OXPHOS occurs in the mitochondrial gyres located in the midpiece. OXPHOS is a significantly more efficient method of ATP production than glycolysis. Despite this, spermatozoa from most heavily researched species, including the human and laboratory rodents, depend predominantly on glycolysis for ATP production [12].

The role of glycolysis in driving the production of ATP for motility has been well researched due to its relative importance in human and laboratory species. Large polar molecules such as glucose cannot diffuse across membranes, and their transport is facilitated by membrane bound proteins called glucose transporters (GLUTs) [13]. GLUTs are categorised according to their relative ability to transport hexoses (such as glucose, mannitol, and fructose), amino sugars, or vitamins [14]. Since the discovery of the glucose transporter GLUT1, many additional GLUTs have been characterised [15, 16]. In spermatozoa of the stallion, GLUTs are localised to the tail and acrosome, suggesting that glycolytic processes are involved in generating energy for the membrane modifications required for capacitation and the acrosome reaction [16]. In glycolytic spermatozoa, the distribution of GLUTs changes along with the capacitation status of the cell (i.e., between noncapacitated and capacitated states) to provide energy at the sites requiring membrane modifications or hyperactivation of motility [16]. In contrast, the distribution of GLUTs on stallion spermatozoa does not change with the capacitation status of the cell [16], indicating that, in species who rely on OXPHOS, glycolysis is not required to support ATP production for motility, capacitation, or the acrosome reaction.

Despite the well-characterised presence of GLUTs on equine sperm, it has become abundantly evident that stallion spermatozoa differ from that of other well-studied mammalian species, in that their energy demands are met not by glycolytic pathways but by using OXPHOS [17–19], and in the presence of mitochondrial inhibitors, they suffer a rapid loss of velocity and a dramatic decline in ATP content [17]. This dependence results in a nonconventional relationship between ROS production and fertility in the stallion [17–19], with the source of ROS being the mitochondrial electron transport chain, in which about 1–3% of O_2_ reduced in the mitochondria during OXPHOS forms superoxide [20].

There is a long-standing paradigm that it is the nonviable or poor quality spermatozoa that generate the most ROS [21]. An alternative explanation is that rapidly metabolising spermatozoa from highly fertile stallions exhibit higher levels of OXPHOS activity, following* in vitro* storage prior to AI present with elevated levels of ROS generation and lipid peroxidation. Thus, while human clinical data steadily report negative correlations between male fertility and sperm oxidative stress [22, 23], a recent study has revealed a paradoxical inverse relationship between fertility and the percentage of live cells* without* oxidative damage in the stallion [17]. In addition, more fertile ejaculates (those which resulted in a pregnancy following insemination) had lower vitality and a higher percentage of cells displaying ROS-induced damage following* in vitro* storage compared to ejaculates which did not result in a pregnancy [17]. From these results, it was hypothesised that during* in vitro* storage spermatozoa from the more fertile stallions (assumed to be more metabolically active) were becoming exhausted more rapidly, such that, by the time that the assays were performed in the laboratory, these cells had suffered an accelerated demise due to the accumulation of metabolic by-products, such as ROS and cytotoxic lipid aldehydes in a “live fast-die young” paradigm. Another interesting observation was that the greater efficiency of OXPHOS mediated ATP production by equine spermatozoa supported a higher velocity, with stallion spermatozoa being around 60% faster than human spermatozoa. Ultimately, high ROS production by stallion spermatozoa appears to be a physiologically normal scenario brought about by superoxide leakage from the mitochondrial electron transport chain during OXPHOS [18], with a positive relationship between mitochondrial ROS production and sperm velocity, leading to increased rates of lipid peroxidation [17] and, following prolonged storage, a loss of motility and vitality [24]. This phenomenon introduces a number of implications for the* in vitro* storage of stallion spermatozoa, since the prolonged generation of ROS in the absence of extracellular free radical and lipid aldehyde scavengers will lead to irreversible oxidative damage, impairing DNA integrity and sperm functionality.

## 3. *In Vitro* Storage of Spermatozoa

In the horse, the most common reason for sperm storage prior to AI is the asynchronous nature of ovulation in the mare. This makes it difficult to predict the precise time of ovulation [25] and means that stored spermatozoa must retain their functionality and longevity for extended periods to allow for the possibility of a delayed ovulation. The long-term storage of spermatozoa is useful so that AI may be performed when ovulation is deemed imminent (based on follicle size determined via transrectal ultrasonography). If AI is to be performed within 12 h of semen collection, spermatozoa are generally left at room temperature (“fresh”). If sperm longevity must be maintained for longer periods, spermatozoa are either chilled (up to 72 h) or cryopreserved (indefinite) to restrict the metabolic rate of the spermatozoa. This temperature-induced metabolic restriction reduces the rates of both ROS production and acidification of the storage medium through the accumulation of lactic acid and CO_2_ from glycolysis and OXPHOS, respectively. However, the spermatozoa of many stallions, and indeed men, do not tolerate the stressors associated with chilling or cryopreservation [26–29]. Therefore, there is a need to develop a medium which will extend the longevity of spermatozoa without the need to chill or be cryopreserved.

## 4. Sperm Cryopreservation

Cryopreservation is presently the only viable method of* in vitro* storage of spermatozoa for periods exceeding 72 h. However, the process of cryopreservation and thawing reduces the acrosomal integrity, viability, and motility of spermatozoa in all species examined including the horse [30–32] while for human spermatozoa there is evidence that cryopreservation results in the formation of DNA lesions on genes that are essential for fertilisation and normal embryonic development [33]. Many of the deleterious effects induced by cryopreservation may be attributed to osmotic stress. During cooling below 0°C, extracellular ice crystals begin to form. This phase change causes a large increase in the osmolarity of any remaining liquid to which the spermatozoa are exposed, placing cells under extreme osmotic stress [34, 35]. Additionally, the cryoprotectants make the cryodiluent hyperosmotic, which causes dehydration of the cells through osmosis [36]. While this dehydration is essential for postthaw viability to be maintained, the extreme hyperosmolarity induces cellular stress as water rushes across the sperm membrane via water channels in an attempt to balance the osmolarity [37]. The result of these osmotic stressors includes membrane damage [38], DNA damage [39, 40], and the production of ROS which causes premature capacitation-like changes [41].

## 5. Chilling

The Standardbred, Sport Horse, and Polo Pony industries are almost entirely dependent on AI for breeding purposes. Sperm chilling is the most widely utilised technique for the transport and storage of stallion spermatozoa. “Chilling” is most commonly achieved using commercial passive cooling devices which slowly cool extended semen to a temperature of between 4 and 10°C, an adequately low enough temperature to restrict metabolism sufficiently to maintain acceptable sperm functionality for up to 72 h. However, stallion spermatozoa are significantly more susceptible to cold shock than those of other species, probably due to a lower ratio of cholesterol to phospholipid in the sperm membranes [42] and, as a result, the insemination of chilled semen is associated with success rates as low as 44% per cycle [3]. As with cryopreservation, there are significant unexplained differences between stallions in the suitability of their semen for low temperature storage [43], a phenomenon which reduces the commercial viability of such animals due to sperm damage following chilling [44]. Additionally, animal derived compounds, such as milk and egg yolk, are routinely incorporated into media for chilled and frozen semen due to their membrane-stabilising effects [45]. This presents a major biosecurity concern for customs authorities and, as such, is a chief limiting factor for the genetic improvement of herds in geographically isolated countries such as Australia.

## 6. Ambient Temperature Storage

The development of a medium that allows spermatozoa to be stored at ambient temperatures for at least one week would permit the importation of new genetics into geographically isolated areas, while avoiding the loss of fertility that occurs following semen chilling and cryopreservation. Moreover, as ambient temperature storage does not require the addition of animal-derived products, such as egg yolk and skim milk for membrane stabilisation, the biosecurity risks associated with importing spermatozoa will be considerably reduced.

There are several implications that arise when higher temperatures are utilised for the* in vitro* storage of stallion spermatozoa. The first of these is the growth of bacteria in the nutrient rich semen extender during storage. Many microbes are present on the penis of the stallion; these include normal commensal bacteria along with microbes from soil, water, and faeces which may contaminate the penis when the stallion gallops, rolls, or lies down in the paddock. Through the process of semen collection using an artificial vagina, these bacteria will inevitably contaminate the ejaculate [46]. Several of these strains have been shown to be deleterious to sperm motility and vitality, even during chilled storage at 4°C [47] and following cryopreservation [48]. However, several antibiotic formulations have been investigated for their effects on curtailing bacterial growth in extended stallion semen [47, 49], and based on these studies, further work in our laboratory has revealed that a storage medium containing 0.25 mg/mL gentamicin, 50 *µ*g/mL streptomycin, and 50 IU/mL penicillin is able to suppress the growth of bacteria for up to one week at room temperature (Gibb et al. unpublished data).

**Figure 1:**
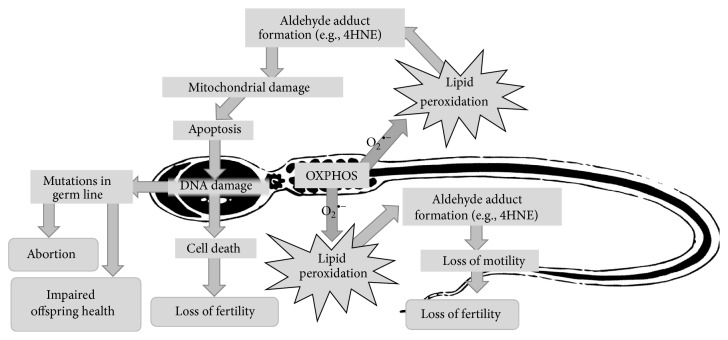
Implications of oxidative phosphorylation (OXPHOS) on sperm storage* in vitro*. Mitochondrial superoxide (O_2_^•−^) leakage causes lipid peroxidation and reactive electrophilic aldehyde production. These aldehydes adduct to functional proteins resulting in motility loss and mitochondrial damage, which may trigger apoptosis and oxidative DNA damage. If this damage does not result in cell death, then germ line mutations may cause embryonic failure and abortion or, should the mutations not be lethal, result in poor health in the resulting offspring.

If sperm metabolism is not restricted by temperature reduction, OXPHOS will produce significant quantities of ROS [20], which will compromise sperm function [11, 24] (Figure 1). The majority of attempts to assuage the damaging effects of ROS on stallion spermatozoa through antioxidant supplementation either have produced marginal improvements [50–54] or have had detrimental effects [55, 56]. This is in contrast to the positive effects seen in human spermatozoa [57, 58] and may be due in part to their alternative mode of ATP production. More recently, the antioxidant properties of carnitine have come into the spotlight [59–63]. L-Carnitine supplementation of stallion spermatozoa during* in vitro* storage significantly reduces both mitochondrial free radical production and lipid peroxidation [63], suggesting that the beneficial effects observed by others may well be attributed to L-carnitine's antioxidant properties. However, supplementation with L-carnitine alone does not completely abolish ROS-induced damage, indicating that it is insufficient for the complete scavenging of ROS [63]. Clearly, further refinement of the antioxidants that might be used to facilitate the long-term storage of stallion spermatozoa is required. Given that mitochondrial metabolism is the source of the majority of ROS, a mitochondrial antioxidant that will also act as a regulator of mitochondrial bioenergetic functions may present the best option to reduce the downstream effects of ROS on sperm function and DNA integrity. While L-carnitine meets these requirements, it is possible that at 50 mM we have reached its beneficial limits and that supplementation with additional antioxidants capable of performing alternative roles in mitochondrial energy production homeostasis may be necessary. Possible candidates for this role are coenzyme Q_10_, an integral component of the electron transport chain and an antioxidant capable of counteracting the ROS-induced peroxidation of mitochondrial phospholipids [64], and melatonin, a free radical scavenger which reduces nitric oxide generation within mitochondria while performing bioenergetic functions by regulating respiratory complex activities, Ca^2+^ influx, and mitochondrial permeability transition pore opening [65].

Sperm motility is lost as a consequence of lipid peroxidation not only due to ROS attack [66], but also due to the concomitant depletion of ATP [67] which compromises myriad ATP-dependent functions, disrupting homeostasis and hastening cell death [68]. If spermatozoa are to be stored at ambient temperatures, it is vital to support mitochondrial energy production while reducing avoidable ATP depletion which results when pressure is placed on ATP-dependent pathways such as the regulation of ionic flux [69]. By utilising nonionic, organic osmolytes, such as betaines, carbohydrates, and amino acids, in place of sodium chloride, pressure on the ATP-dependent Na^+^/K^+^ pumps is alleviated, deducing the rate of ATP depletion [69]. Recent research has revealed that supplementing media with pyruvate, the primary energy source utilise for OXPHOS, and L-carnitine, the biologically active free form of carnitine which plays an essential role in mitochondrial ATP synthesis while being a powerful antioxidant [70] and an organic, nonionic osmolyte [71], results in the maintenance of motility and viability at room temperature akin to that of chilled semen for up to 72 h [63]. Furthermore, stallion spermatozoa contain a number of proteins involved in beta-oxidation of mitochondrial fatty acids and inhibition of this metabolic pathway leads to reduced motility, indicating its significance in fertility [72]. As L-carnitine plays an essential role in beta-oxidation, in addition to its role as an antioxidant and osmolyte, it boosts mitochondrial ATP production through the transportation of acetyl groups from pyruvate into the mitochondrial matrix and through the buffering of free CoA. The acetylation of carnitine (acetyl-L-carnitine; ALCAR) by spermatozoa occurs across the outer mitochondrial membrane to facilitate the provision of acetyl groups for *β*-oxidation and entry into the citric acid cycle for ATP production. The* in vivo* importance of L-carnitine in sperm quality is well recognized [73–78]. Androgen regulated epithelial cells actively secrete L-carnitine into the epididymal lumen [79, 80] resulting in concentrations of up to 2000-fold higher than that of blood, with spermatozoa containing the highest intracellular concentrations of L-carnitine in the body [76], suggesting that this molecule is of extreme importance in fertility. In addition, oral supplementation of L-carnitine results in increased uptake of pyruvate by spermatozoa [81], demonstrating an important interactive role between these compounds in the support of sperm metabolism.

## 7. Conclusion

As the major implication for ambient temperature storage of equine spermatozoa is the production of ROS as a consequence of OXPHOS, future research should concentrate on reducing the deleterious effects of this pathway either through the redirection of metabolism towards the less deleterious glycolysis or through elucidating the mechanisms behind the reversible mitochondrial uncoupling which induces a quiescent state during the* in vivo* storage of spermatozoa in the epididymis. Work in our laboratory has revealed that mouse epididymal fluid can reversibly uncouple sperm mitochondria [82], a phenomenon which we have also observed in the horse. Once this factor has been identified, there is the potential to exploit it to induce sperm quiescence during* in vitro* storage akin to that in the epididymis. In addition, we have found that supplementation of sperm storage medium with rosiglitazone, a member of the thiazolidinedione family of compounds, significantly enhances sperm longevity during storage at ambient temperature. We hypothesize that rosiglitazone is redirecting stallion sperm metabolism from OXPHOS to glycolysis by increasing the efficiency of glucose uptake through GLUT1 [83] and the preferential utilisation of both aerobic [84] and anaerobic glycolysis [85]. While currently the only feasible method for the indefinite storage of spermatozoa is cryopreservation, methods for the storage of spermatozoa at room temperature for at least one week, a sufficient period of time to account for the logistical constraints surrounding insemination and IVF protocols, are in the final stages of optimization. This development will make the need to chill spermatozoa obsolete and in many cases will also negate the need to cryopreserve for gamete importation purposes, resulting in improved per-cycle fertility and embryo development rates.
